# Senile Gangrene1Read at the annual meeting of the Medical Society of Erie County, January 12, 1892.

**Published:** 1892-03

**Authors:** Herman Mynter

**Affiliations:** Buffalo, N. Y.; Professor of Operative and Clinical Surgery, Niagara University; 195 Franklin Street


### SENILE GANGRENE.[1]

By HERMAN MYNTER, M. D.,
Buffalo, N. Y.
Professor of Operative and Clinical Surgery, Niagara University.

SENILE gangrene is always caused by arterio-sclerosis. Senile marasmus, in the form of atheromatous degeneration, may occur in persons under forty years of age and be absent in octogenarians. I have myself seen a case of complete gangrene of one leg in a woman thirty-five years of age in which no other cause could be found.

Holmes states, too, that a person may be prematurely old from excess and suffer from gangrene at a comparatively early age. He mentions several cases, the youngest being twenty-three years.

An absolute vegetable diet is said to be followed by atheromatous degeneration of the arteries. (Holmes.) The same statement is made by Gubler, who believes that the calcareous degeneration of the arteries in pure vegetarians is due to the greater excess of earthy mineral salts in vegetables than in animal food. An exclusive diet of vegetables introduces an excess of these salts into the blood. Similar observations have been made by Raymond, who saw numerous cases of atheroma in a monastery of vegetarian friars. The prior, for instance, thirty-two years of age, had well-marked arterio-sclerosis.

Reports of many similar cases come from Bombay and Calcutta in people who live on an exclusive diet of rice.

A Dr. Alanus, who had lived for a long time as a vegetarian, made the discovery (published in the *Rhenish Courier*) that his arteries were becoming atheromatous, particularly noticeable in the temporal and radial arteries. He was below forty years of age, and could not account for it until he saw the connection between atheroma and a vegetable diet traced in a work by Monin, of Paris, in which Gubler is quoted. Dr. Alanus promptly returned to a mixed diet. In how far the frequent occurrence of atheromatous degenerations in diabetic patients may be traced to their preference for starchy, *i. e.*, vegetable food, future researches will show. It is a well-known fact that diabetic patients are apt to suffer from diffuse and localized inflammations and from gangrene, which is in no way different from senile gangrene. Examination for sugar ought never to be neglected in patients with gangrene, so much more is an antidiabetic diet apt to arrest the gangrene, if diabetes is present. I saw such a case a week ago, being called in consulta-

1. Read at the annual meeting of the Medical Society of Erie County, January 12, 1892.

tion to see a Catholic priest with a large inflammatory swelling in the middle of the back. The swelling was actually as large as a dinner-plate, and presented the common appearance of an enormous carbuncle. My first question was about diabetic symptoms. They had not been inquired into, but he suffered, nevertheless, from extreme thirst and frequent and copious urination. The urine had a specific gravity of 1030, and contained a great amount of sugar.

König found that sugar diminished in the urine after amputation for diabetes.

Israel, also, has pointed out the frequency of arterio-sclerosis in diabetes, finding it in thirteen out of twenty cases. As gangrene very rarely occurs in youthful patients with diabetes, the arterio-sclerosis is probably the main cause of the gangrene. The gangrene in diabetes may occur spontaneously or follow suppuration. König considers senile gangrene the result of an inflammatory stasis, which follows a slight injury, coldness and numbness having existed for some time previously. According to him, it is rarely the result of a marastic thrombus in the terminal vessels, producing localized mummification without previous inflammation, still more rarely the result of an embolus or of primary thrombosis of a large artery.

Heidenhain, on the other hand, who reports thirty cases of amputation from Küster's Clinic in the September number, 1891, of *Deut. Med. Wochensch.*, found by the examination of the amputated limbs, as a rule, an old thrombus in the femoral artery or in the anterior or posterior tibial artery. The thrombus was found particularly frequent in the popliteal artery.

Alcoholism is probably more or less a cause of gangrene on account of the fatty degeneration of the heart.

Moore gives as a primary cause, rigidity of the arteries and diminished blood supply on account of osseous deposits and diminished elasticity; as secondary cause slight injury under above circumstances, such as to corns, nails, particularly in cold weather.

Bryant gives as cause direct, arterial obstruction from atheroma or embolic plugging from breaking loose of some portion of the diseased arterial coat. Ergotism may, of course, produce gangrene from paralysis of the muscles in the vessels; so may we find gangrene on account of deficient nerve power in paralysis or in feeble, fatty heart, and if arterio-sclerosis be present as predisposing cause they may probably act as exciting causes. So may slight contusions or wounds, as was the case in three cases of my own. If the supply of blood be insufficient, either on account of thrombus or embolus, we meet the dry gangrene with mummified appearance and lack of exudation. When the veins are obstructed, particularly on account of inflammation, we get copious exudation and moist gangrene. The rapidity of the extension in dry gangrene depends upon the condition of the bloodvessels. The gangrene may attack one toe or many or the whole leg, following one of the large arteries. Constitutional symptoms are absent and a line of demarcation will usually appear.

In moist gangrene, on the other hand, we have a copious exudation and infection with pus-microbes and putrefactive bacteria, which enter through the always present wound. Septicemia is always present, and a line of demarcation rarely forms.

In regard to symptoms, I refer particularly to Senn's Principles of Surgery.

The great *pain* and numbness preceding the gangrene, sometimes for weeks and due to the gradual ischemia, deserves mention, and these symptoms alone may put us on our guard.

*Acute inflammation* resulting in gangrene is attended with intense pain, which abates when gangrene occurs.

*Tenderness* to touch proves that gangrene yet is absent, just as absence of pain by puncture or incisions proves it present. The importance of this symptom is often overlooked. I was shortly ago called to a town some seventy-five miles from Buffalo, the dispatch stating an amputation necessary on account of blood-poisoning. I found a child, five years of age, who, eight days previously, had stepped on a nail. There was complete gangrene of the foot to above the ankle and enormous swelling of the whole leg, his femur being as large in circumference as my own from purulent edema, septic phlebitis and lymphangitis. The local doctor had treated the little sufferer antiphlogistically with calomel, physics and quinine, but had not thought of relieving the strangulation by incising the plantar fascia. Amputation was, of course, out of question. I made a great many long incisions to relieve the tension, but the patient died inside twelve hours.

The *pulse* may be felt above the thrombus, and furnishes the most important means of following the growth of an intravascular clot and determining its place.

The *swelling* is considerable in moist gangrene, and the slough is larger than the tissue it represents. In dry gangrene the parts shrink and there is a diminution in size as compared with the normal condition.

*Emphysema* indicates the presence of different bacteria.

*Color* is first pale, then livid and of a lead-color, where the circulation has been arrested. The colored blood corpuscles undergo disintegration, the coloring material being diffused through the tissues and the interior of the bullæ. The black color is due to sulphuret of iron.

In dry gangrene we have no *odor*, in moist gangrene an offensive smell from putrefactive bacteria. Mummification can only take place where putrefaction is absent, a line of demarcation only in dry gangrene. Back of this line we have a hyperemic zone, due to collateral circulation.

The most important question is that of treatment. All authors agree in trying to prevent threatening gangrene by elevation of the limb and by heat; to prevent putrefaction by antiseptic measures; to prevent stasis by gentle rubbing towards the center and by avoiding everything which could produce stasis, by scrupulous care of abrasions, corns, bunions, etc.; to prevent marasmus by the judicious use of stimulants, tonics and a liberal diet; but when the question of primary amputation comes up we have any number of conflicting opinions, not alone in regard to the advisability of amputation *per se*, but also in regard to the point of election. Nevertheless, the opinions differ only in regard to dry gangrene, as Nature occasionally is able to overcome this unaided. In moist gangrene amputation is recognized as the only hope, and even here the successes have been few and far between.

Holmes says: "Let the surgeon beware of interfering with the separation of slough. Nature will accomplish this task best when unaided and surgical interference is, as a rule, dangerous."

Bryant: "As there is a depressed state of system, general strengthening measures as nutritious food stimulants and tonics are indicated. Amputation at line of demarcation when established."

Agnew: "Senile gangrene does not admit of operative interference. Surgeons are generally content to wait till line of demarcation is formed. There is only one justification for amputation, *i. e.*, line of demarcation, and the amputation must be done as near the ulcerating surface as possible."

Wyeth: "No operative proceeding justifiable until a well-defined line of demarcation is formed, unless septic absorption threatens life."

Moore: "Wait till Nature has arrested the progress of the disease, except in traumatic gangrene after crushing or after operations, which sometimes spread with great rapidity in uninjured parts. In such cases amputate high above the gangrene as it is useless to wait for line of demarcation."

Such opinions might be multiplied *ad infinitum* by consulting all the standard works on surgery in the English language. They all recommend to wait for the line of demarcation, as in that case there is established a compensating circulation and, at least, an attempt of granulation. In the periodical literature, on the other hand, we find numerous cases reported of early and successful cases of amputation in senile gangrene, sufficient to show that this wait-and-do-nothing policy is not adopted as the general rule in the profession. I do not at all deny that occasionally Nature can accomplish wonders, as, for instance, in the case of a patient on Niagara square, in Buffalo, whose leg, under homeopathic treatment, was allowed to amputate itself below the knee and really succeeded in doing so. Mr. Reginald Harrison (*Lancet*, November, 1884,) showed, at the Medical Society in London, a mummified foot and leg separated by spontaneous amputation. The patient was seventy-nine years of age and recovered. It is probably the best policy where only a toe or two are gangrenous and I have carried it out in one case in the General Hospital:

The patient was a man, sixty-one years of age, who had been healthy till three years previously when he lost all the toes and a part of the foot of left leg by senile gangrene, the process lasting fifteen months. The leg was thereafter amputated in middle third below the knee. Some sloughing occurred in the flaps but the wound healed. Six months later gangrene occurred of plantar side of right great toe, which came away in five months at the metatarso-phalangeal joint, and the wound healed. Then the second toe became gangrenous and sloughed away, then the fifth. On entering the hospital the fourth toe was black and mummified at second phalanx; pulse, 104; temperature, 98; no pain. The arteries in the wrist and femur felt like strings of beads. The toe was allowed to slough off and the stump healed.

Nevertheless, this is not the normal course by any means. In the great majority of cases the disease, as acknowledged by the standard authors, will terminate fatally in one to eight weeks, at least, before the line of demarcation has shown itself.

Watson Cheyne advocates, from theoretical reasons just mentioned, early amputation.

Mr. Harrison Cripps (*British Medical Journal* of May, 1885,) reports a case from St. Bartholomew's Hospital, in which he amputated in the lower third of crus. The patient was sixty-five years, diabetic and a drunkard, with slowly progressing gangrene, which had attacked the whole foot. The wound healed without accident.

James Cantlie (*British Medical Journal* of October, 1886,) had a case of recovery in a lady eighty-two years of age, beginning in the great toe. Teale's amputation above the ankle was performed and the wound healed perfectly.

Mr. Keall, of Bristol, (*Lancet*, 1884,) reports a case in a man in which he made a partial amputation, first removing two gangrenous toes and heads of metatarsals from right leg, there being no line of demarcation and the incisions passing through the red, inflamed and edematous integuments of the dorsum of the foot. Eleven months later a Forbes' amputation was performed on left foot. He advocates early operation through moderately healthy tissue.

Hutchinson is the first one who strongly recommends, in all cases of senile gangrene, to amputate the femur in the lower third. In amputation below the knee relapse will occur according to his statement.

König warns against being too ready to operate in diabetic and senile gangrene, unless septicemia is present.

In diabetic gangrene the prognosis has always been considered particularly bad.

Mayer mentions eleven amputations, of which six died and the gangrene returned in the five left. Of sixty-one not operated cases eighty per cent. died.

In *Deutsche Med. Wochenschrift*, of September, 1891, I find a lengthy and very important contribution to the subject by Heidenhain, who reports thirty cases of amputation from Küster's Clinic in twenty-five patients, three patients having double amputations, and one triple amputation. Eleven had diabetes and fourteen pure arterio-sclerosis. Besides these thirty amputations ten secondary amputations were performed on the same patients on account of return of gangrene.

Küster made first low amputations but got relapse every time, and then amputated high up and became, therefore, gradually convinced of the necessity of making high amputations as soon as the gangrene had reached the dorsum or planta of the foot. Of thirteen primary amputations below the knee (including Chopart's and Lisfranc's amputations) only two amputations of the crus healed, two died of gangrene of flaps, and nine were reamputated above the knee. The result of high amputations was much more favorable. Of *eight* primary low femur amputations through the condyles, two died of diabetic coma and six recovered, but in two cases reamputations higher up became necessary on account of gangrene of the flaps. Thirteen supra-condylic amputations (six primary and seven secondary, for recurrent gangrene) are mentioned.

Four died of the *six* primary amputations (one of gangrene of flaps and hypostatic pneumonia, one of marasmus, one of diabetic coma, and one (fifty-eight days later) of recurrent gangrene). Two recovered, but in both gangrene recurred, and one was reamputated in the middle of the femur.

Of the *seven* secondary amputations two healed by first intention, three after slight marginal gangrene, and two after extensive flap gangrene.

To recapitulate, of *seventeen* primary amputations through or above the knee nine recovered and eight died of diabetic coma and heart complications, but of the nine only two healed by first intention, three after marginal gangrene, one after extensive gangrene and three after amputations higher up.

Of the ten secondary femur amputations all recovered, three by first intention, six after marginal gangrene and one after reamputation. *Eleven* of the twenty-five patients had *diabetic gangrene*, of which six recovered by a high amputation and five died, four of coma and one of heart disease. Fourteen had pure senile gangrene, of which nine recovered and five died, two of recurrent gangrene following a Lisfranc amputation and a low crus amputation, two old people (seventy-eight and eighty years old) of hypostatic pneumonia, and one patient of myocarditis and nephritis. All patients recovered by high amputations who did not suffer from diabetes, albuminuria or heart troubles. It is of interest to note that low amputation generally terminated in gangrene. Heidenhain explains this by the usual fund of a thrombus in the femoral or popletial arteries.

I have, in the last few years, had occasion to amputate three times for senile gangrene. Two died, a low crus amputation and a low femur amputation, both from recurrent gangrene, while one recovered from a high femur amputation although very much debilitated at the time.

Case I. was that of a man sixty-three years of age, a grocer and moderate drinker. I was called in consultation by the late Dr. Meisburger and heard the following history:

The patient stepped on a nail three weeks previously and an inflammation beneath the plantar fascia resulted. Some small incisions had been made, but there was diffuse moist gangrene without line of demarcation, extending till above malleolus internus.

Amputation of crus in lower third. The arteries were found as stiff as leadpencils, and were ligated some inches higher up. The gangrene recurred promptly. Reamputation was declined and he died on the seventh day.

I then made up my mind, in a similar case, to amputate higher up.

Case II. A man, sixty-three years of age; rich brewer; hard drinker. I was called in consultation by Dr. Cott. Had for two months intolerable pain in the right big toe, which was livid and had a gangrenous spot as large as a ten-cent piece, opening into the metatarso-phalangeal joint. He ascribed the condition to a bad corn, which a barber had cauterized with caustic potash. I did not suspect senile gangrene and, therefore, amputated the toe and the head of the metatarsal bone. Next day gangrene of the dorsum appeared, reaching on the second day to the malleolus internus. Third day, amputation in lower third of femur, but even here the arteries were moderately sclerotic. Gangrene recurred and he died on the ninth day.

Case III. A man, sixty-four years of age; moderate drinker. He had a slight injury two months previously, followed by inflammation and gangrene, intolerable pain and great weakness. The gangrene extended up along the inner side of the foot to the internal malleolus; no line of demarcation. Amputation in upper part of crus, but, as the arteries were found full of chalky material, amputation in the middle part of femur was made.

He made a good recovery. The wound healed by first intention, except on one spot over the inner part, where a little hardness was felt which increased in size and at last protruded as a bleeding mass through the skin, as large as a goose-egg, and resembling a fungus hematodes. The wound was re-opened and the tumor found to be an aneurism of the end of the femoral artery. It was extirpated, the artery ligated again and the wound healed promptly.

After this experience, and comparing it with the statistics of Heidenhain and others, I think I am justified in all cases of severe senile gangrene, which takes in more than a toe or two, in recommending immediate amputation in the middle, or rather above the middle, of the femur, and in discouraging all attempts of saving parts of limbs by low amputations, of waiting for a line of demarcation and in a general way of doing nothing, trusting to nature to do what it, in the great majority of cases, will not do.

195 Franklin Street.

